# The chromosome-level quality genome provides insights into the evolution of the biosynthesis genes for aroma compounds of *Osmanthus fragrans*

**DOI:** 10.1038/s41438-018-0108-0

**Published:** 2018-11-20

**Authors:** Xiulian Yang, Yuanzheng Yue, Haiyan Li, Wenjie Ding, Gongwei Chen, Tingting Shi, Junhao Chen, Min S. Park, Fei Chen, Lianggui Wang

**Affiliations:** 1 Co-Innovation Center for Sustainable Forestry in Southern China, Nanjing Forestry University, Nanjing, China; 2 College of Landscape Architecture, Nanjing Forestry University, Nanjing, China; 3 State Key Laboratory of Ecological Pest Control for Fujian and Taiwan Crops, Fujian Agriculture and Forestry University, Fuzhou, China; 4 Nextomics Bioscience Institute, Wuhan, China

**Keywords:** Genome duplication, Genome assembly algorithms

## Abstract

Sweet osmanthus (*Osmanthus fragrans*) is a very popular ornamental tree species throughout Southeast Asia and USA particularly for its extremely fragrant aroma. We constructed a chromosome-level reference genome of *O*. *fragrans* to assist in studies of the evolution, genetic diversity, and molecular mechanism of aroma development. A total of over 118 Gb of polished reads was produced from HiSeq (45.1 Gb) and PacBio Sequel (73.35 Gb), giving 100× depth coverage for long reads. The combination of Illumina-short reads, PacBio-long reads, and Hi-C data produced the final chromosome quality genome of *O*. *fragrans* with a genome size of 727 Mb and a heterozygosity of 1.45 %. The genome was annotated using de novo and homology comparison and further refined with transcriptome data. The genome of *O*. *fragrans* was predicted to have 45,542 genes, of which 95.68 % were functionally annotated. Genome annotation found 49.35 % as the repetitive sequences, with long terminal repeats (LTR) being the richest (28.94 %). Genome evolution analysis indicated the evidence of whole-genome duplication 15 million years ago, which contributed to the current content of 45,242 genes. Metabolic analysis revealed that linalool, a monoterpene is the main aroma compound. Based on the genome and transcriptome, we further demonstrated the direct connection between terpene synthases (TPSs) and the rich aromatic molecules in *O*. *fragrans*. We identified three new flower-specific *TPS* genes, of which the expression coincided with the production of linalool. Our results suggest that the high number of *TPS* genes and the flower tissue- and stage-specific *TPS* genes expressions might drive the strong unique aroma production of *O*. *fragrans*.

## Genome sequences: Discovering a sweet secret

A high-quality genome sequence for sweet olive, *Osmanthus fragrans*, has revealed which genes make the ornamental tree’s blossoms so fragrant. Lianggui Wang at Nanjing Forestry University in China and coworkers used cutting-edge approaches to produce a high-resolution picture of sweet olive’s genome. Analysis of the full sequence revealed that sweet olive has a large number of genes, more than 45,000. Further investigation showed that the entire genome had been duplicated, or accidentally copied, several million years ago. Thanks to the duplication, sweet olive has extra copies of the genes that produce its scent compound, resulting in its very strong scent. These results are only the beginning. Having this first sequence makes sequencing other varieties of sweet olive much easier, and future comparisons between genomes will help to pinpoint genes that control many other traits.

## Introduction

Sweet osmanthus (Dicotyledons, Lamiales, Oleaceae, *Osmanthus*) is one of the most popular, evergreen ornamental tree species in China due to its unique sweet aroma^1,2^. More than 160 cultivars of *O*. *fragrans* have been classified based on phenotypes such as the leaf shape, flower color, aroma, season and frequency of flower blooming^3^. The association between phenotypes and genotypes of *O*. *fragrans* has been examined through aroma compounds^4–6^, essential oils^7–10^, and taxonomy using various molecular markers^11–16^. Transcriptome studies have determined the genes that might be responsible for the emission of flower scent in *O. fragrans*^17,18^. Gene expression has also been modulated at different flowering stages of *O*. *fragrans*^19^. Differential gene expression studies have identified genes in the mediated isopentenol production (MEP) pathway, as well as the terpenoid- and carotenoid-synthesis pathways. Transcriptomics studies allowed researchers to make connections between the major flower aroma compounds, and differentially expressed genes and encoded proteins. The flower aroma compounds, (*R*)- and (*S*)-linanool are produced by a terpene synthase(s) (TPS)^20^. Another key flower aroma compound, *β*-ionone, is produced through the oxidative cleavage of *β*-carotene by carotenoid cleavage enzymes (CCD)^21–23^. Transcriptome studies have shown that TPS(s) and CCD(s) are differentially expressed at different flowering stages in *O. fragrans*^24,25^. Additionally, we and others have recently reported a set of transcription factors (TFs) associated with the expression of color and the emission of fragrance in *O. fragrans*^21,26,27^. All of these gene expression studies provide valuable insights on how flower blooming and aroma production are interlinked^28^. However, a genome sequence is largely needed to reveal the full genetic background of aroma production in sweet osmanthus and the evolution of aroma in the family Oleaceae.

In this study, we generated a reference genome for *O. fragrans* to provide a solid foundation for our future understanding of the genome structure and evolution of the Oleaceae family. Furthermore, we conducted a detailed analysis of the aroma compounds, tissue and flowering time-specific differential gene expression to investigate the molecular mechanisms of sweet fragrance development in *O. fragrans*.

## Results

### Sequencing summary

We generated 100-fold PacBio single-molecule long reads (a total of 73.4 Gb with an N50 length of 13.0 kb), 77-fold *k*-mer depth Illumina paired-end short reads (45.1 Gb) and Hi-C data that produced 23 unambiguous chromosome scaffolds for a high-quality assembly. For stepwise assembly, we first performed an initial PacBio-only assembly, resulting in an assembly size of 733.5 Mb and a contig N50 of 1.59 Mb, and the assembled genome had a highly complete BUSCOs (96.1 %) (Supplemental Table 1). Then, the initial contigs were subsequently polished with PacBio long reads and Illumina short reads. As the final step, Hi-C data were used to polish the scaffolds generated by the PacBio and Illumina reads.

### Determination of genome size and heterozygosity

The *k*-mer method^29^ and KmerFreqAR^30^ were used to determine the genome size of *O. fragrans* using the quality-filtered reads of Illumina data. The genome size was estimated based on the formula: Genome size = Modified *k*-mer number/average *k*-mer depth, where modified *k*-mer = Total *k*-mer number−error *k*-mer number and the average *k*-mer depth obtained from the main peak of *k*-mer distribution curve (Supplemental Fig. 1). To determine the heterozygosity, *Arabidopsis* genome data were used to simulate Illumina PE reads, which was carried out by using pIRS software^31^. Then, a fitting KmerFreqAR^30^ was developed using the *k*-mer distribution curve of *O. fragrans*. When the two *k*-mer curves were consistent, the heterozygosity of *Arabidopsis* was considered the reference for the heterozygosity of *O. fragrans*. The final analysis produced ~1.45% heterozygosity of the *O. fragrans* genome.

### Genome assembly and quality assessment

The integrated work-flow of genome assembly is shown (Supplemental Fig. 2). The full PacBio long reads were converted to fasta format. Then, all subreads of genome data were assembled using Falcon v0.3.0^32^ with specific parameters (length-cutoff pr = 8 kb; length-cutoff pr = 9 kb). We used Arrow (https://github.com) to polish the draft genome (G1) to obtain the corrected genome (G2). Then, G2 was polished again by Pilon^33^, which mapped the next-generation sequencing data to G2 with bwa to obtain the twice-corrected genome (G3). The *O. fragrans* genome had high heterozygosity, which led to a G3 size larger than the estimation. To acquire the nonredundant genome, heterozygous and redundant sequences were removed from the corrected genome using Redundans^34^ with the following parameters: heterozygosity = 0.0145 and Sequencing Depth = 86. The nonredundant genome (G4) was ~741 Mb, with a contig N50 size of 1.595 Mb (Table 1). Finally, BUSCO v3.0 analysis^35^ was performed to assess G4 using the embryophyta_odb10 database with default parameters.

**Table 1:** Quality assessment statistics of the assembled genome of *O. fragrans*

Stat type	Contig length	Contig number
N50	1,595,720	145
Longest	8,253,028	1
Total	740,635,307	774
Length > 5 kb	740,625,951	765

**Fig. 1:**
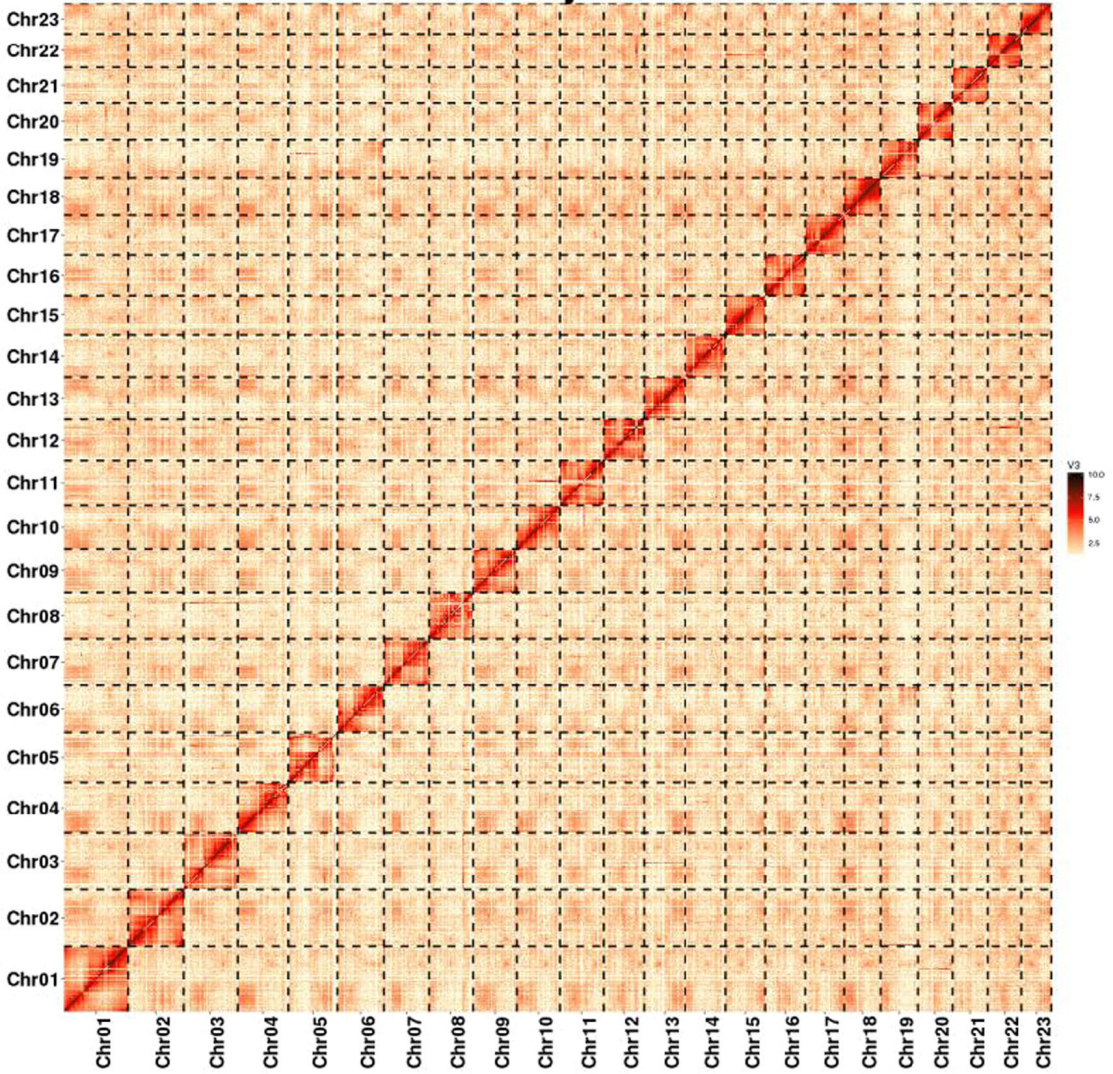
Hi-C map of the O. fragrans genome showing genome-wide all-by-all interactions. The map shows a high resolution of individual chromosomes that are scaffolded and assembled independently

The clustering of contig by hierarchical clustering of the Hi-C data was performed. Through a comparative analysis, the only pair of reads around the DpnII digestion site was determined. Hi-C linkage was used as a criterion to measure the degree of tightness of the association between different contigs by standardizing the digestion sites of DpnII on the genome sketch. Agglomerative hierarchical clustering and LACHESIS produced chromosome assembly maps with a karyotype of 2*n* = 46 (Fig. 1). As a result, the total number of contigs of the *O. fragrans* genome map was 5327, and the total length was 740,635,307 bp. The combined length of Hi-C contigs was 740,404,543 bp, accounted for 99.97 % of the total length of the final assembled genome, indicating the high quality of Hi-C data (Table 2).

**Table 2:** Summary statistics demonstrating the high quality of the Hi-C map of *O. fragrans*

Sample	*O. fragrans*
Draft contig total number	5327
Draft contig total length	740,635,307
Final contig number	5305
Final contig total length	740,404,543
Contig coverage on full genome (%)	99.97

### Annotation of repeat sequences

The genome of *O. fragrans* had simply, moderately, and highly repetitive sequences. MIcroSAtellite was used to identify the repeat sequences in the genome of *O. fragrans* (MISA, RRID: SCR 010765). A total of 409,691 SSRs was obtained, including 305,868 mono-, 70,587 di-, 25,544 tri-, 3934 tetra-, 2081 penta-, and 1991 hexa-nucleotide repeats, respectively (Supplemental Tables 2–3). The tandem repeats finder (TRF, v4.07b)^36^ identified over 400,000 tandem repeats, accounting for 0.076 % of the *O*. *fragrans* genome.

We used homology-based and de novo approaches to identify transposable elements. RepeatMasker^37^ was used to search against the Repbase (v. 22.11)^38^ and Mips-REdat libraries^39^. Then, we used RepeatMasker v4.0.6 to search the de novo repeat library that we built using RepeatModeler v1.0.11 (RepeatModeler, RRID: SCR 015027). Finally, TEs were confirmed by searching the TE protein database using a RepeatProteinMask and WU-BLASTX. The repetitive sequence was 49.35 %, of which LTR accounted for 28.49 % of the assembled genome of *O. fragrans* (Supplemental Table 4).

### Annotation of noncoding RNA (ncRNA)

We identified rRNA, miRNA, and snRNA genes in the *O*. *fragrans* genome by searching the Rfam database (release 13.0)^40^, using BLASTN^41^ (*E*-value ≤ 1e−5). Software tRNAscan-SE (v1.3.1)^42^ and RNAmmer v1.2^43^ were used to predict tRNAs and rRNAs, resulting in an *O*. *frangrans* genome with 525 miRNAs, 847 tRNAs, 49 rRNAs, and 2058 snRNAs (Supplemental Table 5).

### Gene prediction

The protein-coding genes were identified using homology-based and de novo predictions-based approaches. The *O. fragrans* genome was mapped against the published sequences of *Arabidopsis thaliana*, *Olea europaea*, *Sesamum indicum*, *Solanum tuberosum*, and *Vitis vinifera*. To accurately identify spliced alignments, we used GeneWise v2.2.0^44^ to filter all initially aligned coding sequences. For de novo prediction, the data from NGS and the full-length transcriptomes were analyzed with hisat2-2.1.0 and PASApipeline-2.0.2 to predict the complete gene set. We randomly selected 1000 genes to train the model parameters for Augustus v3.3^36^, GeneID v1.4.4^45^, GlimmerHMM^46^, and SNAP^47^. The final consensus gene set was generatedusing EVidenceModeler (EVM) v1.1.1^48^, which combined the genes predicted by the de novo and homology searches^49,50^ The assembled genome had 45,542 genes with an average transcript length of 4065 bp, an average CDS length of 1142 bp, and a number of exons per gene of 5 (Supplemental Table 6).

The functional validity of the predicted genes was further evaluated by searching the UniProt (release 2017_10), KEGG (release 84.0), and InterPro (5.21-60.0) databases using Blastall44, KAAS,49, and InterProScan50. As a result, we were able to assign potential functions to 43,573 protein-coding genes out of the total of 45,542 genes in the *O*. *fragrans* genome (95.68 %) (Supplemental Table 7).

### Genome evolution

#### Gene family analysis

**Fig. 2:**
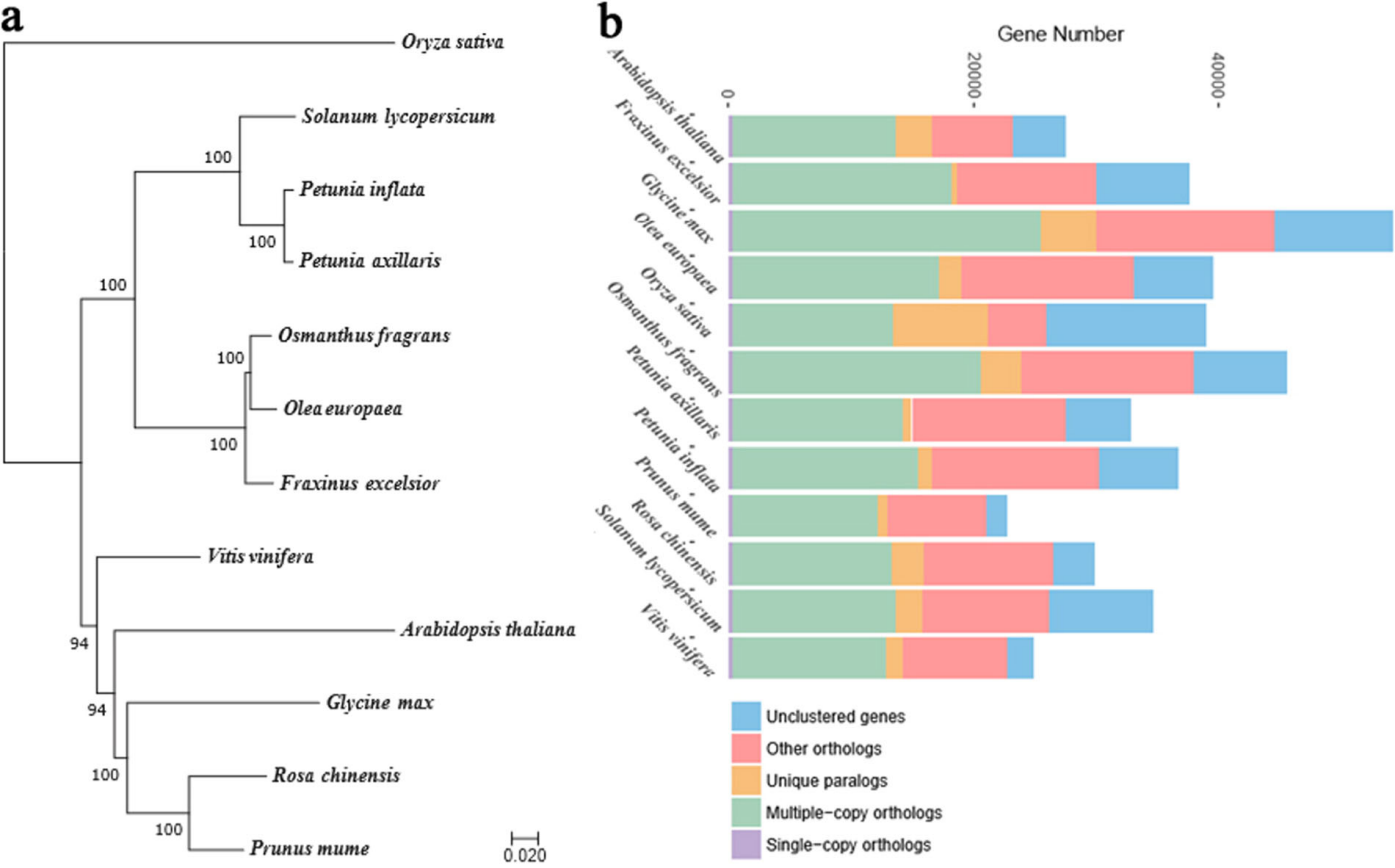
Species tree and evolution of gene numbers. **a** The phylogenetic tree showing the close relationship between sweet osmanthus and the wild olive (*O*. *europaea*). **b** The number of genes in various plant species, showing the high gene number of *O. fragrans* compared to a model (*A*. *thaliana*) and other tree species. The number of multiple copy paralogs is high in *O. fragrans*

Although morphological investigation and a number of genes have placed *O*. *fragrans* in the Oleaceae family, there is still no whole genome-scale phylogenomic analysis of the evolutionary position of *O. fragrans*. Here, we compared the *O. fragrans* genome with the genome sequences of 11 other plants (*A. thaliana, Fraxinus excelsior, Glycine max, O. europaea, Oryza sativa, Petunia axillaris, Petunia inflata, Prunus mume, Rosa chinensis, Solanum lycopersicum*, and *V*. *vinifera)*. We applied the OrthoMCL (v2.0.9) pipeline^51^ (BLASTP *E*-value ≤ 1e−5) to identify the potential orthologous gene families between the genomes of these plants. Gene family clustering identified 17,513 gene families consisting of 38,808 genes in *O*. *fragrans*, of which, 1086 gene families were unique to *O*. *fragrans*. *O*. *europaea*, and *F*. *excelsior* had the biggest number of shared gene families among these plants (Fig. 2).

#### Synteny analysis

**Fig. 3:**
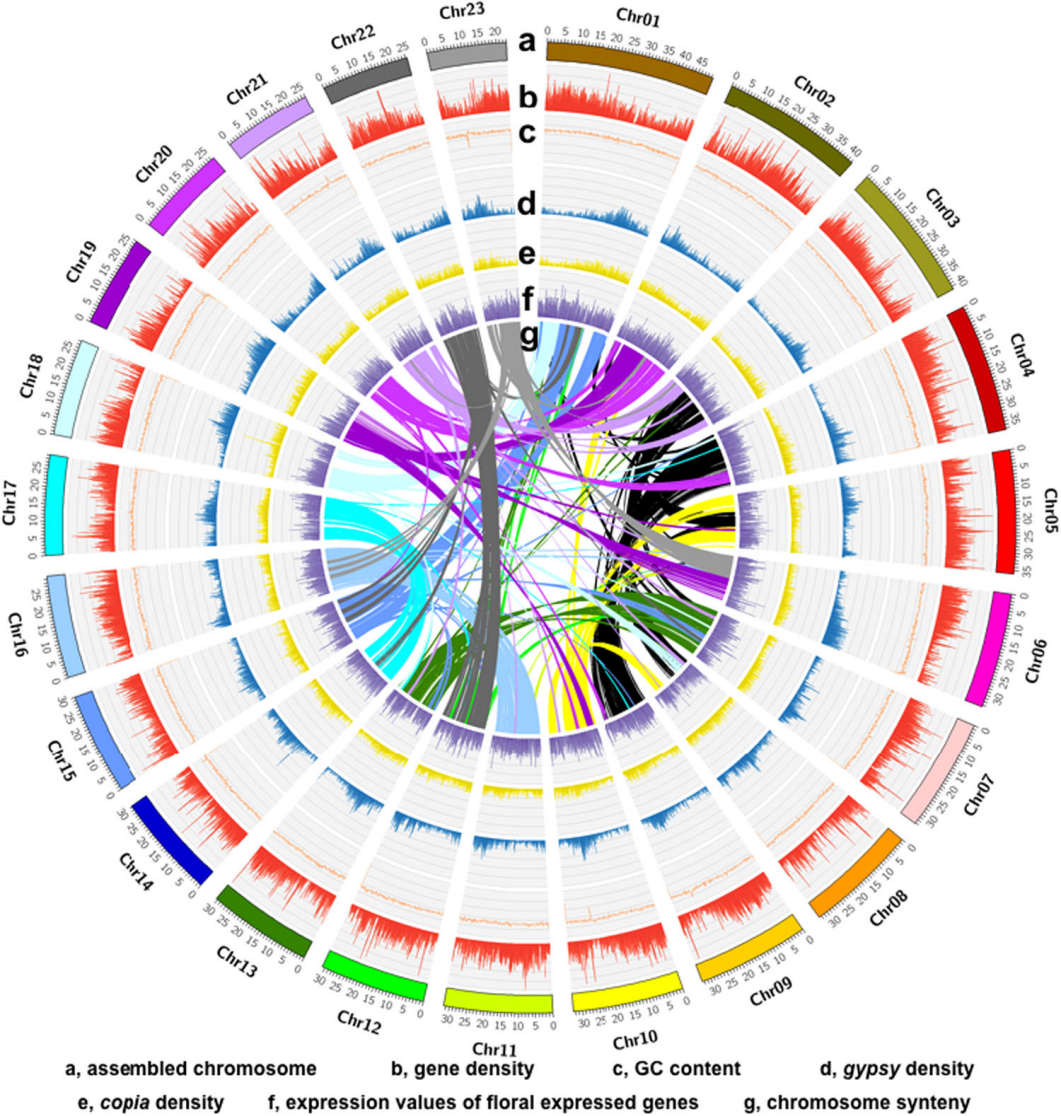
High-quality assembly of twenty-three chromosomes. **a** Gene density, **b** GC content, **c** Gypsy density, **d** Copia density, and **e** average expression values of genes specifically expressed in flowers (from outside to inside)

We used the protein sequences of *O. fragrans* that were aligned against each other with Blastp (*E*-value ≤ 1e−5) to achieve the conserved paralogs, Then, MCScanX (http://chibba.pgml.uga.edu/mcscab2) was used to find the collinearity block in the genome. Using the Circos tool (http://www.circos.ca), we mapped and gene density, GC content, Gypsy density, and Copia density, as well as the average expression value of genes expressed in flowers on individual chromosomes (Fig. 3).

#### Whole-genome duplication (WGD)

**Fig. 4:**
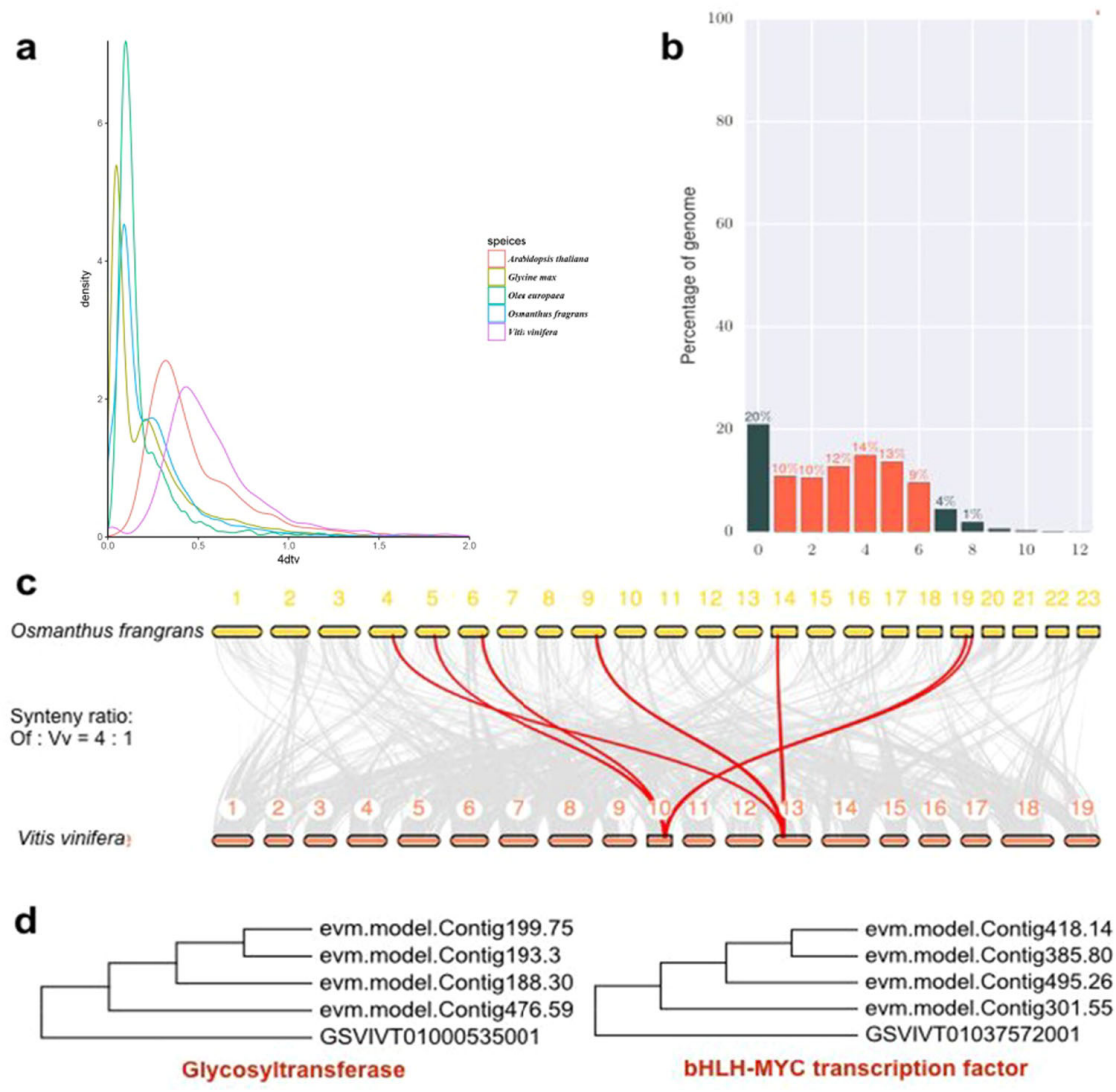
Evidences for whole-genome duplication events in O. fragrans. **a** 4DTV; **b** the most abundant genes (red bar: duplicated genes, green: non-duplicated, 0: tandem-duplicated or small-scale-duplicated genes; 0–12, number of duplicated copies) in a four-fold relationship when comparing *O. fragrans* and grapes. **c** A colinear relationship at the chromosome level. **d** Four-fold expansion of the functional homologs of glycosyltransferase and bHLH-MYC genes in grape (GSVIVT) and *O. frgrans* (evm.model.Contig). The data suggest that *O. fragrans* experienced two WGD events

To determine the source of the high number of genes (>45,000) in *O. fragrans*, the WGD events were analyzed by taking advantage of the high-quality genome of *O. fragrans*. We applied four-fold synonymous third-codon transversion (4DTv) and synonymous substitution rate (Ks) estimation to detect the WGD events. First, respective paralogous of *O. fragrans*, *G. max*, *O*. *europaea*, *V*. *vinifera*, and *A*. *thaliana* were identified with OrthoMCL. Then, the protein sequences of these plants were aligned against each other with Blastp (*E*-value ≤ 1e−5) to achieve the conserved paralogs of each plant. Finally, the WGD events of each plant were evaluated based on their 4DTv (Fig. 4a) or Ks (data not shown) distribution. The WGD analysis suggestted that *O*. *fragrans*, *G*. *max* and *O*. *europaea* experienced WGD events within less than 15 MYA, but *V*. *vinifer* and *A*. *thaliana* have not experienced WGD events recently (Fig. 4a). We also compared the number of duplicated genes (Fig. 4b), the chromosome-level duplications (Fig. 4c), and the number of a functional homologs of glycotransferase and bHLH-Myc transcription factor genes between *O*. *fragrans* and *V*. *vinifera* (Fig. 4d), further validating the WGD events.

### Determination of volatile aroma compounds

To make a direct connection between the biosynthetic genes and flower fragrance development, we determined the volatile aroma compounds. Headspace-SPME combined with GC-MS analysis identified over 40 volatile compounds, including linalool, dihydrojasmone lactone (2(3H)-furanone, 5-hexyldihydro-), 1-cyclohexene-1-propanol, 2,6,6-tetramethyl-, and *β*-ocimen as the major components. Linalool was present in the highest amount at the early flowering stage (S1) and decreased afterwards (Table 3).

**Table 3:** Identity and quantity of volatile aroma compounds in the various flowering stages of *O. fragrans*

Fragrance_molecules	S1(A)	*P*-value(A&B)	S2(B)	*P*-value(B&C)	S3(C)	*P*-value(A&C)
Linalool	6.67 ± 1.63	0.001	2.91 ± 2	0.007	0.22 ± 0.15	0.000
2(3 H)-Furanone, 5-hexyldihydro-	2.58 ± 0.62	0.000	1.29 ± 0.28	0.000	—	0.000
1-Cyclohexene-1-propanol, à,2,6,6-tetramethyl-	2.29 ± 0.63	0.000	0.01 ± 0.01	0.975	—	0.000
β-Ocimene	1.28 ± 0.42	0.002	0.68 ± 0.26	0.001	0.02 ± 0.02	0.000
2-Furanmethanol, 5-ethenyltetrahydro-α,α,5-trimethyl-, cis-	1.25 ± 0.58	0.213	0.82 ± 0.21	0.010	1.79 ± 0.77	0.123
trans-Linalool oxide (furanoid)	1.18 ± 0.57	0.296	0.78 ± 0.25	0.001	2.34 ± 0.91	0.007
(3 R,6 S)-2,2,6-Trimethyl-6-vinyltetrahydro-2H-pyran-3-ol	0.7 ± 0.26	0.050	0.41 ± 0.11	0.003	0.9 ± 0.29	0.162
α-Ionone	0.5 ± 0.18	0.000	0.18 ± 0.1	0.448	0.13 ± 0.04	0.000
2-Butanone, 4-(2,2-dimethyl-6-methylenecyclohexyl)-	0.37 ± 0.13	0.000	0.01 ± 0	1.000	0.01 ± 0	0.000
(3 R,6 R)-2,2,6-Trimethyl-6-vinyltetrahydro-2H-pyran-3-ol	0.35 ± 0.13	0.038	0.2 ± 0.05	0.000	0.51 ± 0.15	0.033
2H-Pyran-3(4 H)-one, 6-ethenyldihydro-2,2,6-trimethyl-	0.33 ± 0.11	0.276	0.22 ± 0.06	0.052	0.43 ± 0.28	0.342
Butanoic acid, 3-hexenyl ester, (E)-	0.14 ± 0.05	0.284	0.1 ± 0.07	0.891	0.1 ± 0.06	0.346
2,4,6-Octatriene, 2,6-dimethyl-, (E,E)-	0.1 ± 0.03	0.055	0.06 ± 0.02	0.253	0.08 ± 0.04	0.387
Methyl salicylate	0.08 ± 0.08	0.881	0.07 ± 0.06	0.046	—	0.034
NA1	0.08 ± 0.03	0.016	0.05 ± 0.02	0.002	—	0.000
3-Buten-2-one, 4-(2,6,6-trimethyl-1-cyclohexen-1-yl)-	0.06 ± 0.02	0.000	1.18 ± 0.58	0.892	1.15 ± 0.28	0.000
NA3	0.06 ± 0.01	0.002	0.03 ± 0.02	0.000	—	0.000
3-Hexen-1-ol, acetate, (Z)-	0.05 ± 0.03	0.899	0.04 ± 0.04	0.003	0.4 ± 0.31	0.004
cis-3-Hexenyl isovalerate	0.05 ± 0.02	0.224	0.03 ± 0.02	0.030	0.06 ± 0.02	0.278
trans-β-Ocimene	0.05 ± 0.01	0.002	0.03 ± 0.01	0.000	—	0.000
Methyl isovalerate	0.04 ± 0.03	0.376	0.03 ± 0.01	0.898	0.03 ± 0.02	0.314
(E)-4,8-Dimethylnona-1,3,7-triene	0.04 ± 0.02	0.046	0.02 ± 0	0.011	—	0.000
Hexanoic acid, methyl ester	0.03 ± 0.02	1.000	0.03 ± 0.01	0.011	—	0.011
cis-3-Hexenyl-α-methylbutyrate	0.03 ± 0.01	0.091	0.02 ± 0.01	0.091	0.03 ± 0.01	1.000
3-Buten-2-ol, 4-(2,6,6-trimethyl-2-cyclohexen-1-yl)-, (3E)-	0.03 ± 0.01	0.000	0.01 ± 0.01	0.119	—	0.000
Octanoic acid, methyl ester	0.02 ± 0.01	0.121	0.01 ± 0	0.592	0.01 ± 0.01	0.045
Tridecane	0.02 ± 0.01	0.015	0.01 ± 0	0.291	0.01 ± 0.01	0.121
trans-β-Ionone	0.02 ± 0.01	0.015	0.01 ± 0	0.000	—	0.000
Megastigma-4,6(Z),8(E)-triene	0.02 ± 0.01	0.000	—	1.000	—	0.000
2-Butanone, 4-(2,6,6-trimethyl-1-cyclohexen-1-yl)-	0.02 ± 0.01	0.010	0.28 ± 0.13	0.001	0.67 ± 0.23	0.000
(3E,7E)-4,8,12-Trimethyltrideca-1,3,7,11-tetraene	0.02 ± 0	0.173	0.03 ± 0.01	0.000	—	0.000
Hexadecane	0.01 ± 0.01	0.072	0.01 ± 0	0.000	—	0.000
Butanoic acid, methyl ester	—	0.000	0.03 ± 0.01	0.000	—	1.000
1-Butanol, 2-methyl-, acetate	—	1.000	—	0.000	0.06 ± 0.03	0.000
NA2	—	1.000	—	0.000	0.01 ± 0.01	0.000
Dodecane	—	1.000	—	0.000	0.02 ± 0.01	0.000
NA4	—	1.000	—	0.000	0.02 ± 0.01	0.000
2H-Pyran-3-ol, 6-ethenyltetrahydro-2,2,6-trimethyl-, acetate, trans-	—	1.000	—	0.000	0.02 ± 0.01	0.000
1-Cyclohexene-1-ethanol, 2,6,6-trimethyl-	—	0.000	0.01 ± 0	0.000	—	1.000

### Expression analysis

**Fig. 5:**
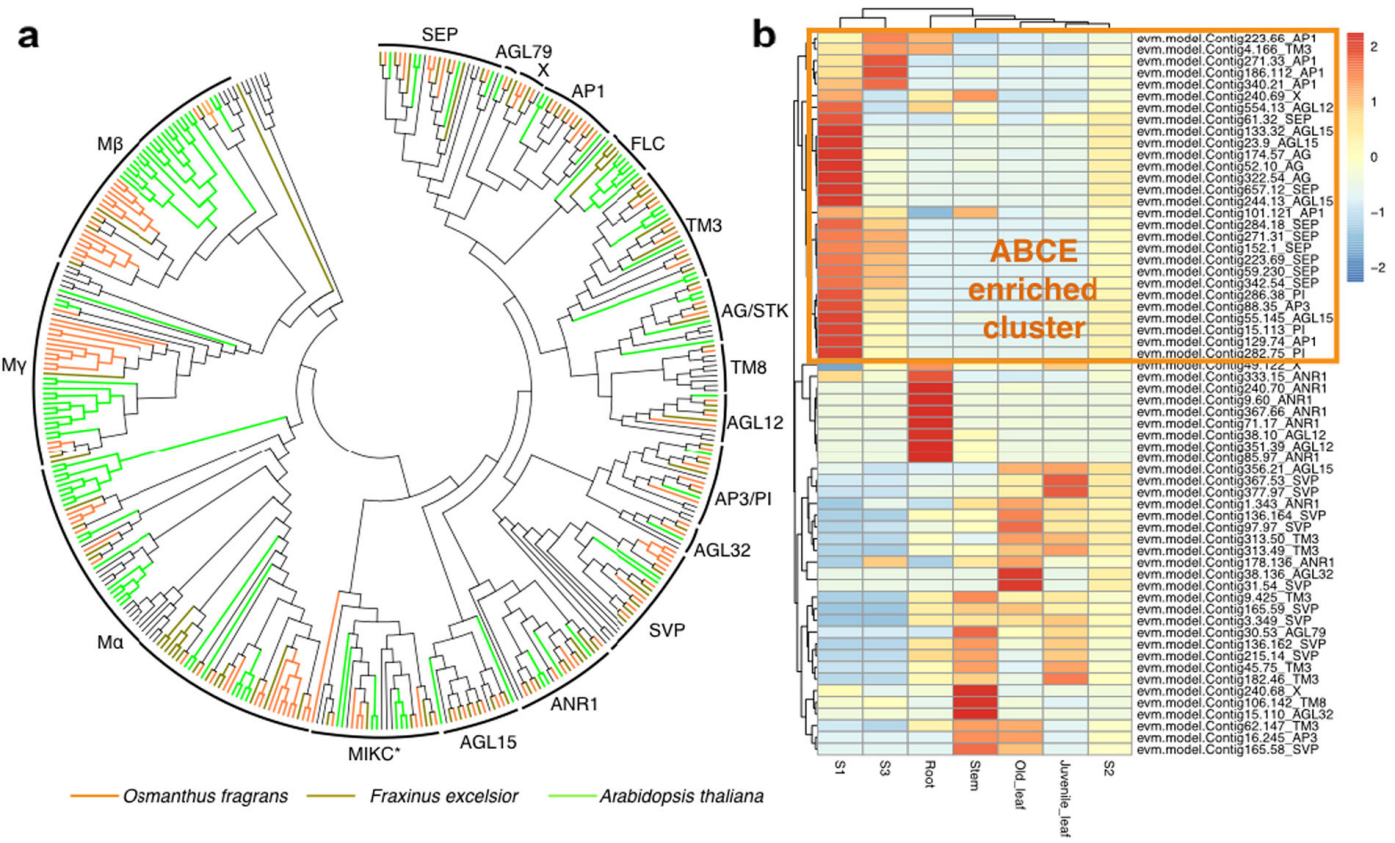
MADS-box gene family in O. fragrans. **a** The evolution tree and expression values of the MADSs box genes, **b** Heatmap showing the tissue- and flowering stage- specific expressions of the members of ABCE genes

We also produced comprehensive transcriptome dataset using both HiSeq and the Iso-Seq pipeline. We focused our further analysis on identifying the specific genes responsible for floral development and the biosynthesis of volatile aroma compounds in *O. fragrans*. The members of MADs transcription factors that control plant development were highly expressed in all tissues tested. Among them, AG, AP3/PI, AP1, and SEP were predominantly expressed in the early flower stage (S1), whereas, the expression level of the *ANR1* gene family was highly specific to the root tissue (Fig. 5b). Interestingly, the numbers of ABCE genes were higher than that of *Fraxinus chinensis*, a close relative of *O*. *fragrans* (Fig. 5a).

**Fig. 6:**
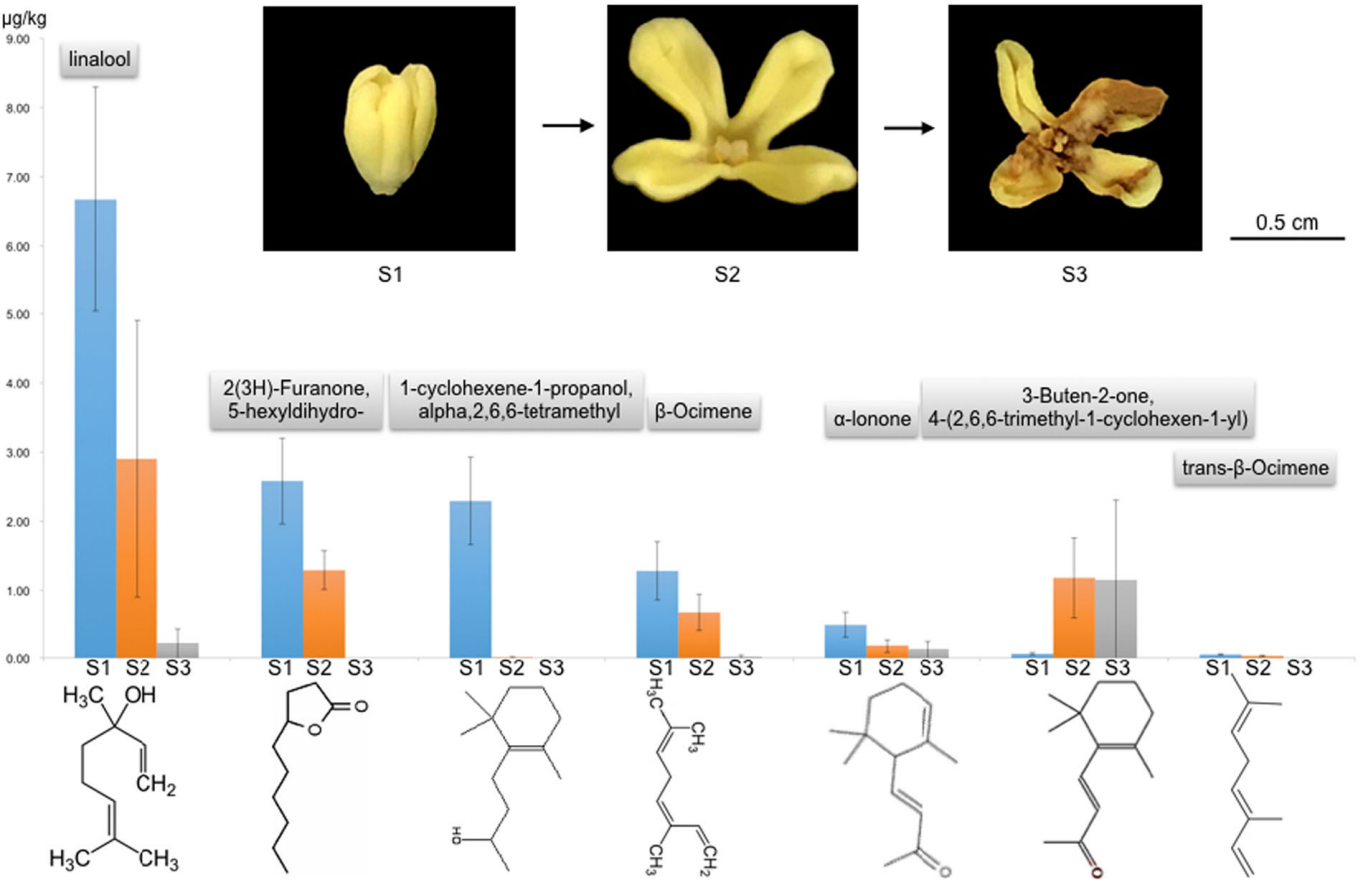
The top 7 secondary metabolites produced by the osmanthus flower measured by GC-MS. Note that the top molecule is linalool, a monoterpene, which was produced most in the S1 stage of the flower, and then decreased its amount in the flower. S1, S2, and S3 stand for the early, middle, and late stages, respectively

**Fig. 7:**
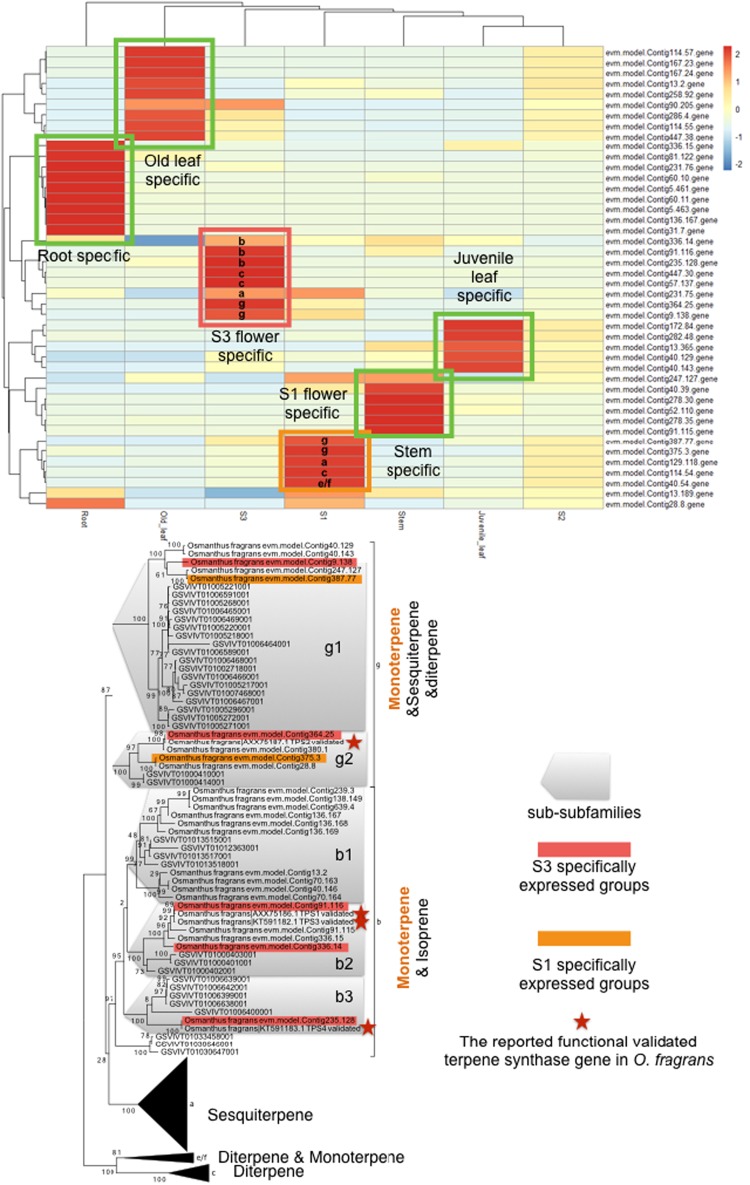
TPS (terpene synthase) gene family in O. fragrans. **a** Heatmap showing the tissue- and flowering stage-specific expression of the members of TPS genes, **b** TPS gene subfamily members. Yellow: S1 early flowering stage-specific Red: S3, late flowering stage-specific

The major component of the volatile compounds in the floral scent of *O*. *fragrans*, linalool (Fig. 6), is known to be synthesized by terpenoid synthetases (TPS). Therefore, we compared the expression profiles of *TPS* genes and identified over 40 genes that contain the functional motifs of TPS. Differential gene expression (DGE) analysis identified 7 *TPS* genes that are highly expressed in flowers, compared to roots, leaves, and stems (Fig. 7).

## Discussion

Sweet osmanthus is one of the most beloved ornamental tree species in China and other parts of the world and has been cultivated for over two-thousand years in China due to its attractive traits of beautiful colors, unique aromas, a long flowering season, and medicinal efficacy. However, there is a limited number of studies that have investigated the genetic basis of the phenotypic diversity of sweet osmanthus. Recently, a set of genetic markers was identified^14,15^, and an effort to construct a genetic linkage map was reported^16^. Additionally, several transcriptomics studies identified a large number of genes that are differentially expressed in some of the cultivars with attractive traits^17,18^. While these studies indirectly associate the diverse phenotypes with the genotypes of sweet osmanthus, there is no genome information that can directly link the specific genes to particular traits. Thus, we have sequenced, assembled and annotated the genome of sweet osmanthus. Furthermore, combining HiSeq- and IsoSeq-based transcriptome analyses, we gained deep insight into the genes that control aroma compounds synthesis in the flowers of *O*. *fragrans*.

### The high-quality reference genome provides deep insights to the evolution of *O*. *fragrans*

Currently, there are still no comprehensive analyses combining genomic, transcriptomic, and metabolic approaches to reveal the unique aroma of *O*. *fragrans*. Despite advances in second-generation sequencing, it is still very challenging to construct a high-quality plant genome due to the high complexity, large size, and high percentage of repeats and polyploidy. Therefore, we combined the second-generation short read to achieve high accuracy, the third-generation long reads for de novo assembly, and Hi-C to scaffold contigs into a chromosome-scale assembly. To guarantee a high-quality genome annotation, we combined *de novo*, homology-based, and experimental evidence obtained from the extensive transcriptomics data, including the full-length transcripts. We constructed a reference-quality genome that produced an unambiguous chromosome-scale assembly (*N* = 23) and functionally annotated 43,573 genes out of the complete set of 45,542 genes of *O. fragrans* (95.68 %).

The number of genes, 45,542, is high and is more than the genes present in some of the plants that are related to *O. fragrans* (Fig. 2). This can be attributed to the repeated gene duplications which led to expansion of the gene families. The *O. fragrans* genome has higher number of multicopy genes compared to other plant species (Fig. 2). Furthermore, *O*. *fragrans* appears to have obtained and retained a large number of genes through the whole genome duplications (Fig. 4). The majority of plant species have experienced genome duplications in their evolutionary past^52,53^. The high gene number of *O. fragrans* might be a result of complex interactions among various factors such as the rate of evolution, number of duplication events, level of gene retention, expansion of gene family and selection pressure. The recent (~15 MYA) WGD and high retention might explain the large gene number. The number of genes involved in secondary metabolism is particularly high in *O. fragrans* (unpublished observation), and these genes might have been retained and/or expanded after the whole-genome duplications. This result may reflect the continuous interaction between *O. fragrans* and environmental factors, which imposes a constant pressure for adaptation^54^.

The calculated level of heterozygosity (1.45 %) is high in *O. fragrans* var ruixiangui. Considering that *O. fragrans* has been selectively bred for desirable traits for over 2000 years in China, 160 cultivars with diverse phenotypes have been selected. The high heterozygosity (1.45 %) in *O. fragrans* var ruixiangui might support an extensive breeding among cultivars throughout its history, although it is challenging to accurately determine the origin of the observed heterozygosity in the cultivar. Furthermore, as an androdioecious species^55^, the coexistence of selfing and crossing poses an additional challenge to trace the origin of the high heterozygosity. Recently, the first genetic map of *O. fragrans* was created using the SLAF-seq method^16^ to provide a framework for understanding the genome organization. This linkage map has helped us assemble the reference genome and can help to investigate the origin of the high heterozygosity and history of hybridization among the cultivars of *O*. *fragrans*.

The new genome can also be used as a reference for the whole genome resequencing of sweet osmanthus cultivars. Resequencing these whole genomes of various cultivars provides highly useful information on the potential drivers for the phenotype diversity, evolution, and population structure of a given species^56^. Our preliminary genome sequencing of 30 different cultivars of *O. fragrans* identified a large number of single nucleotide polymorphisms (SNP), copy number variation (CNV), insertion sdeletion (InDel), structural variations (SV) and other mutation sites (unpublished results). Using the above mutation loci as new molecular genetic markers, researchers can study the history of cultivation, population dynamics and genetic diversity.

### The whole-genome duplication and the tandem duplication of the biosynthetic genes is likely the cause for the strong sweet aroma of *O. fragrans*

Among *TPS*-family genes, the *TPS-b* and *g* subfamilies are known to synthesize monoterpenes^57^. Linalool, the major aroma compound identified in our study, is produced by the monoterpene synthesis pathway in *O. fragrans*. Using the high-quality genome and deep transcriptome information, we found a significant expansion of TPS as a whole, and of subfamilies b and g specifically, compared with the grape (*V*. *vinifera*), which did not have whole genome duplication (Fig. 5). In addition to TPS1, 2, 3, and 4, which have been previously functionally validated, we identified seven additional *TPS* genes that are specifically expressed in flower stages S1 and S3. Three *TPS* genes appear to be new genes that are flower specific, indicating that the production of fragrance is controlled by a complex network involving multiple *TPS* genes functioning in time- and flower-specific manners. Our results suggest that the unique aromas of *O. fragrans* are some of the outcomes of the interrelationship between genome evolution, transcriptional regulation, and metabolic control. Our current work lays a solid foundation for further studies on the comparative genomics, molecular and biochemical mechanisms of aroma development in *O*. *fragrans*.

## Conclusion

We constructed a high-quality reference genome of *O*. *fragrans* by combining Illumina, PacBio and Hi-C platforms. The genome of *O*. *fragrans* var. rixianggui is ~740 Mb and has a high heterozygosity of 1.45 %. A large number of genes (45,542) was predicted by the gene models built with de novo, homology-based, and experimental data obtained from extensive transcription results. Our deep genome analysis indicates evidence of whole-genome duplication at ~15 MYA. Our new genome information should help the research community study the genome structure, genetic basis of genetic diversity, and regulation of the flowering process and scent development in *O. fragrans* and other related plant species.

## Materials and methods

### Genome sequencing

For genome sequencing, leaf samples were collected from a male tree (*O*. *fragrans* var. rixianggui) on the campus of Nanjing Forestry University, Nanjing, China, and were processed for genomic DNA isolation and library construction. Rixianggui (Semperfloren) is a unique cultivar because it has a strong aroma and blooms continuously, except in hot summer months, while other cultivars, for example, Thunbergii, Latifolius, and Aurantiaeus, bloom only in autumn. Genomic DNA was extracted using the CTAB method, size fractionated with BluePipin (Sage Science, Inc, MA, USA), used for library construction following the PacBio SMRT library construction protocol, and sequenced on the PacBio Sequel platform (Pacific Biosciences, CA, USA). For Illumina library construction, the extracted DNA was fragmented and size-fractionated using g-tube and BluePipin, then subjected to paired-end library construction and sequenced on the HiSeq X ten platform (Illumina Inc, CA, USA).

### Hi-C sequencing

To ensure the quality of the Hi-C library, leaf samples were initially examined forintegrity of the nuclei by DAPI staining. Once confirmed for high quality nuclei, the samples were processed following the Hi-C procedure^58–60^. The Hi-C library was sequenced on the Illumina HiSeq X ten platform (Illumina, CA, USA), generating 740 million Hi-C read pairs, which were submitted to the Lachesis Hi-C scaffolding pipeline^58^. Hi-C libraries produce different molecular types, including invalid pairs of self-circles, dangling-ends, and dumped-pairs. According to the different molecular types that lead to the alignment of paired reads on the genome in different directions, the unique alignment of reads on the genome needs to be statistically analyzed. Once recognized as an effective interaction, the final data only retained effective interactions. According to the above rules, the position of the DpnII digestion site in the reference genome was used, because it can also provide useful information on the structural organization of individual chromosomes.

### Transcriptome sequencing

To obtain information that can assist in the empirical annotation of genes, full-length transcriptome sequencing was performed. The samples from flowers at three different blooming stages (S1: beginning, S2: middle, S3: late; Suppl. Figure 3), leaves, stems, and roots were collected from the same tree described above and processed for library construction. The total RNAs were extracted according to the manufacturer’s instructions of TRNzol Universal Reagent (Cat# DP424, TIANGEN Biotech Co. Ltd, Beijing, China). The quality and quantity of the RNA samples were evaluated using a NanoDrop™ One UV-Vis spectrophotometer (Thermo Fisher Scientific, USA), a Qubit® 3.0 Fluorometer (Thermo Fisher Scientific, USA) and an Agilent Bioanalyzer 2100 (Agilent Technologies, USA). All RNA samples with integrity values close to 10 were used for cDNA library construction and sequencing. The cDNA library was prepared using the TruSeq Sample Preparation (Illumina Inc, CA, USA) and IsoSeq Library Construction kits (Pacific Biosciences, CA, USA), and paired-end sequencing with 150 bp was conducted on a HiSeq X ten platform (Illumina Inc, CA, USA).

### Aroma compound analysis

Fresh flowers at three different stages (S1: beginning, S2: middle, S3: lat), defined by the size of the flower (Supplemental Fig. 3), were picked from the same tree at the time of sample collection for the transcriptome studies described above. Sampling was replicated five times, and the samples were quickly put into polyethylene bags impermeable to gases, kept frozen and stored at −20 °C. Headspace solid phase microextraction (SPME) combined with gas chromatography-mass spectrometry (GC-MS) was used to determine the identity and quantity of the aroma volatiles. Flowers (0.3 g) were placed in a 4 mL solid-phase microextraction vial (Supelco Inc, USA), 1 μl of 1000× diluted ethyl caprate (Macklin Inc, China) was added, and vials were capped with a 65 µm DB-5 ms extraction head (Supelco Inc, USA). Then, the vial was incubated for 40 min in a water bath at 45 °C to volatilize the aroma compounds and release them into the headspace. After the adsorption period, the fiber head was removed and introduced into the heated injector port of the GC for desorption at 250 °C for 3 min. The desorbed volatile compounds from DB-5 ms were analyzed on a Trace DSQ GC-MS (Thermo-Fisher Scientific, USA), equipped with a 30 m x 0.25 mm × 0.25 mm TR-5 ms capillary column (Supelco Inc, USA). The oven temperature was programmed at 60 °C for 2 min, increasing at 5 °C/min to 150 °C, then increasing at 10 °C/min to reach 250 °C, followed by maintaining the temperature of the transfer line at 250 °C. Helium was taken as the carrier gas at a linear velocity of 1.0 mL/min. Mass detector conditions on MS were: source temperature: 250 °C and the electronic impact (EI) mode at 70 eV, with a speed of 4 scans/s over the mass range m/z 33-450 amu in a 1 s cycle. Volatile compounds were first auto-matched by mass spectra using the NIST98 database through ChemStation (Agilent, USA). A series of *n*-alkanes (C7-C30) (Sigma St. Louis, MO) was injected into the GC-MS set to obtain the linear retention indices of the volatile compounds, and they were analyzed under the same conditions. The data were also compared with published linear retention indices (NIST Chemistry WebBook, SRD 69). The normalization of peak-areas was used to calculate the quantities of the volatile aroma compounds.

## Electronic supplementary material

new Supplemental

## References

[1] Shang FD, Yin YJ, Xiang QB (2003). The culture of sweet osmanthus in China. J. Henan Univ. Nat. Sci..

[2] Hao RM, Zang DK, Xiang QB (2005). Investigation on natural resources of *Osmanthus fragrans* Lour. at Zhou luo cun in Hunan. Acta Hortic. Sin..

[3] Zang DK, Xiang QB, Liu YL, Hao RM (2003). The studying history and the application to International Cultivar Registration Authority of sweet osmanthus (*Osmanthus fragrans* Lour.). J. Plant Resour. Environ..

[4] Deng CH, Song GX, Hu YM (2004). Application of HS-SPME and GC-MS to characterization of volatile compounds emitted from Osmanthus flowers. Ann. Chim..

[5] Xin HP (2013). Characterization of volatile compounds in flowers from four groups of sweet osmanthus (*Osmanthus fragrans*) cultivars. Can. J. Plant Sci..

[6] Cai X (2014). & W, C.Y. Analysis of aroma-active compounds in three sweet osmanthus (*Osmanthus fragrans*) cultivars by gas-chromatography olfactometry and GC-mass spectrometry. J. Zhejiang. Univ. Sci. B.

[7] Hu CD (2009). Essential oil composition of *Osmanthus fragrans* varieties by GC-MS and heuristic evolving latent projections. Chromatographia.

[8] Wang LM (2009). Variations in the components of *Osmanthus fragrans* Lour. Essential oil at different stages of flowering. Food Chem..

[9] Hu BF, Guo XL, Xiao P, Luo LP (2012). Chemical composition comparison of the essential oil from four groups of *Osmanthus fragrans* Lour. flowers. J. Essent. Oil Plants.

[10] Lei GM (2016). Water-soluble essential oil components of fresh flowers of *Osmanthus fragrans* lour. J. Essent. Oil Res..

[11] Shang FD, Yin YJ, Zhang T (2004). The RAPD analysis of 17 *Osmanthus fragrans* cultivars in Henan province. Acta Hortic. Sin..

[12] Yuan WJ, Han YJ, Dong MF, Shang FD (2011). Assessment of genetic diversity and relationships among *Osmanthus fragrans* cultivars using AFLP markers. Electron. J. Biotechnol..

[13] Hu W, Luo Y, Yang Y, Zhang ZY, Fan DM (2014). Genetic diversity and population genetic structure of wild sweet osmanthus revealed by microsatellite markers. Acta Hortic. Sin..

[14] Yuan WJ, Li Y, Ma YF, Han YJ, Shang FD (2015). Isolation and characterization of microsatellite markers for *Osmanthus fragrans* (Oleaceae) using 454 sequencing technology. Genet. Mol. Res..

[15] Han YJ (2015). cDNA-AFLP analysis on 2 Osmanthus fragrans cultivars with different flower color and molecular characteristics of MYB1gene. Trees.

[16] He, Y. X., Yuan, W. J., Dong, M. F., Han, Y. J. & Shang, F. D. The first genetic map in sweet osmanthus (Osmanthus fragrans Lour.) using specific locus amplified fragment sequencing. *Front. Plant Sci.* **8**, 1621 (2017). 10.3389/fpls.2017.01621 PMC5614988 29018460

[17] Zhang XS, Pei JJ, Zhao LG, Tang F, Fang XY (2018). RNA-Seq analysis and comparison of the enzymes involved in ionone synthesis of three cultivars of Osmanthus. J. Asian Nat. Prod. Res..

[18] Yang XL (2018). Transcriptomic analysis of the candidate genes related to aroma formation in *Osmanthus fragrans*. Molecules.

[19] Xu C (2016). Cloning and expression analysis of MEP pathway enzyme-encoding genes in *Osmanthus fragrans*. Genes.

[20] Zeng XL (2015). Emission and accumulation of monoterpene and the key terpene synthase (TPS) associated with monoterpene biosynthesis in *Osmanthus fragrans* Lour. Front. Plant Sci..

[21] Baldermann S (2010). Functional characterization of a carotenoid cleavage dioxygenase 1 and its relation to the carotenoid accumulation and volatile emission during the floral development of *Osmanthus fragrans* Lour. J. Exp. Bot..

[22] Baldermann S, Kato M, Fleischmann P, Watanabe N (2012). Biosynthesis of *α*- and *β*-ionone, prominent scent compounds, in flowers of *Osmanthus fragrans*. Acta Biochim. Pol..

[23] Han YJ, Liu LX, Dong MF, Yuan WJ, Shang FD (2013). cDNA cloning of the phytoene synthase (PSY) and expression analysis of PSY and carotenoid cleavage dioxygenase genes in *Osmanthus fragrans*. Biologia.

[24] Han YJ (2014). Differential expression of carotenoid-related genes determines diversified carotenoid coloration in flower petal of *Osmanthus fragrans*. Tree. Genet. Genom..

[25] Zhang C, Wang YG, Fu JX, Bao ZY, Zhao HB (2016). Transcriptomic analysis and carotenogenic gene expression related to petal coloration in *Osmanthus fragrans* ‘Yanhong Gui’. Trees.

[26] Mu HN (2014). Transcriptome sequencing and analysis of sweet osmanthus (*Osmanthus fragrans* Lour.). Genes. Genom..

[27] Han YJ (2016). Characterization of *OfWRKY3*, a transcription factor that positively regulates the carotenoid cleavage dioxygenase gene *OfCCD*4 in *Osmanthus fragrans*. Plant Mol. Biol..

[28] Wang L (2017). Analysis of the main active ingredients and bioactivities of essential oil from *Osmanthus fragrans* Var. thunbergii using a complex network approach. BMC Syst. Biol..

[29] Guillaume M, Carl K (2011). A fast, lock-free approach for efficient parallel counting of occurrences of K-Mers. Bioinformatics.

[30] Luo R (2012). SOAPdenovo2: an empirically improved memory-efficient short-read de novo assembler. Gigascience.

[31] Hu X (2012). pIRS: Profile-based Illumina pair-end reads simulator. Bioinformatics.

[32] Chin CS (2016). Phased diploid genome assembly with single-molecule real-time sequencing. Nat. Methods.

[33] Walker BJ, Abeel T, Shea T, Priest M, Abouelliel A, Sakthikumar S, Cuomo CA, Zeng Q, Wortman J, Young SK, Earl AM (2014). Pilon: an integrated tool for comprehensive microbial variant detection and genome assembly improvement. PLoS One.

[34] Pryszcz LP, Gabaldón T (2016). Redundans: an assembly pipeline for highly heterozygous genomes. Nucleic Acids Res..

[35] Simão FA, Waterhouse RM, Ioannidis P, Kriventseva EV, Zdobnov E (2015). BUSCO: assessing genome assembly and annotation completeness with Single-Copy Orthologs. Bioinformatics.

[36] Stanke M, Steinkamp R, Waack S, Morgenstern B (2004). AUGUSTUS: a web server for gene finding in eukaryotes. Nucleic Acids Res..

[37] Chen N (2004). Using RepeatMasker to identify repetitive elements in genomic sequences. Curr. Protoc. Bioinforma..

[38] Bao W, Kojima KK, Kohany O (2015). Repbase Update, a database of repetitive elements in eukaryotic genomes. Mob. DNA.

[39] Nussbaumer T (2013). MIPS PlantsDB: a database framework for comparative plant genome research. Nucleic Acids Res..

[40] Kalvari I (2017). Rfam 13.0: shifting to a genome-centric resource for non-coding RNA families. Nucleic Acids Res..

[41] Camacho C (2009). BLAST+: architecture and tapplications. BMC Bioinforma..

[42] Lowe TM, Eddy SR (1997). tRNAscan-SE: a program for improved detection of transfer RNA genes in genomic sequence. Nucleic Acids Res..

[43] Lagesen K, Hallin P, Rødland EA, Staerfeldt HH, Rognes T (2007). RNAmmer: consistent and rapid annotation of ribosomal RNA genes. Nucleic Acids Res..

[44] Birney E, Durbin R (2000). Using GeneWise in the Drosophila annotation experiment. Genome Res..

[45] Blanco E, Parra G, Guigó R (2007). Using geneid to identify genes. Curr. Protoc. Bioinforma..

[46] Majoros WH, Pertea M, Salzberg SL (2004). TigrScan and GlimmerHMM: two open source ab initio eukaryotic gene-finders. Bioinformatics.

[47] Bromberg Y, Rost B (2007). SNAP: predict effect of non-synonymous polymorphisms on function. Nucleic Acids Res..

[48] Haas BJ (2008). Automated eukaryotic gene structure annotation using EVidenceModeler and the program to assemble spliced alignments. Genome Biol..

[49] Moriya Y, Itoh M, Okuda S, Yoshizawa AC, Kanehisa M (2007). KAAS: an automatic genome annotation and pathway reconstruction server. Nucleic Acids Res..

[50] Quevillon E (2005). InterProScan: protein domains identifier. Nucleic Acids Res..

[51] De Bodt S, Maere S, Van de Peer Y (2005). Genome duplication and the origin of angiosperms. Trends Ecol. Evol..

[52] Li L, Stoeckert CJ, Roos DS (2003). OrthoMCL: identification of ortholog groups for eukaryotic genomes. Genome Res..

[53] Ciu LY (2006). Widespread genome duplications throughout the history of flowering plants. Genome Res..

[54] Casneuf T, De Bodt S, Raes J, Maere S, Van de Peer Y (2006). Nonrandom divergence of gene expression following gene and genome duplications in the flowering plants Arabidopsis thaliana. Genome Biol..

[55] Xu YC (2014). The differentiation and development of pistils of hermaphrodites and pistillodes of males in androdioecious Osmanthus fragrans L. and implications for the evolution to androdioecy. Plant Syst. Evol..

[56] Huang X (2009). High-throughput genotyping by whole-genome resequencing. Genome Res..

[57] Tholl D (2006). Terpene synthases and the regulation, diversity and biological roles of terpene metabolism. Curr. Opin. Plant. Biol..

[58] Kaplan N, Dekker J (2013). High-throughput genome scaffolding from in vivo DNA interaction frequency. Nat. Biotechnol..

[59] Marie-Nelly H (2014). High-quality genome (re) assembly using chromosomal contact data. Nat. Commun..

[60] Jibran R (2018). Chromosome-scale scaffolding of the black raspberry (Rubus occidentalis L.) genome based on chromatin interaction data. Hortic. Res..

