# Supplementary material for: The chromosome-level quality genome provides insights into the evolution of the biosynthesis genes for aroma compounds of Osmanthus fragrans
Source: Hortic Res. 2018 Nov 20;5:72. doi: 10.1038/s41438-018-0108-0 (PMC6246602; doi:10.1038/s41438-018-0108-0)
Supplement: Supplementary file 1 — new Supplemental [file 41438_2018_108_MOESM1_ESM.docx]

**Supplemental Table 1 Quality assessment of the assembled genome of *O*. *fragrans* using BUSCOs.**

| **Type** | **Number** | **Percent (%)** |
| --- | --- | --- |
| Complete BUSCOs (C) | 1,322 | 96.1 |
| Complete and single-copy BUSCOs (S) | 935 | 68.0 |
| Complete and duplicated BUSCOs (D) | 387 | 28.1 |
| Fragmented BUSCOs (F) | 35 | 2.5 |
| Missing BUSCOs (M) | 18 | 1.4 |
| Total BUSCO groups searched | 1,375 | 100 |

**Supplemental Table 2 Statistics of the annotated simple sequence repeats (SSR) in the *O*. *fragrans* genome.**

|  | | **Number / Size** |
| --- | --- | --- |
| Total examined sequences | 768 / 740,633,826 | |
| Total identified SSR | 409,691 | |
| Compound format | 62,549 | |
| Sequences contain SSR | 765 | |
| Sequences contain SSR (>1) | 765 | |

**Supplemental Table 3 Specific statistics of the annotated SSRs in the *O*. *fragrans* genome.**

| **Type** | **Unit size**  **(repeat number)** | **Number** |
| --- | --- | --- |
| p1 | 1(>=10) | 305868 |
| p2 | 2(>=6) | 70587 |
| p3 | 3(>=5) | 25544 |
| p4 | 4(>=5) | 3949 |
| p5 | 5(>=5) | 2081 |
| P6 | 7(>=5) | 1662 |

| \| **Type** \| **Repbase TEs** \| \| **TE proteins** \| \| **Repeat Modeler** \| \| **Combined TEs** \| \| \| --- \| --- \| --- \| --- \| --- \| --- \| --- \| --- \| --- \| \| **Length**  **(Mb)** \| **% in genome** \| **Length**  **(Mb)** \| **% in genome** \| **Length**  **(Mb)** \| **% in genome** \| **Length**  **(Mb)** \| **% in genome** \| \| DNA \| 11466986 \| 1.55 \| 13854578 \| 1.87 \| 35500978 \| 4.79 \| 42348260 \| 5.72 \| \| LINE \| 2324325 \| 0.31 \| 4764469 \| 0.64 \| 5344922 \| 0.72 \| 6954170 \| 0.94 \| \| LTR \| 96988128 \| 13.1 \| 99688493 \| 13.46 \| 156097224 \| 21.08 \| 210982390 \| 28.49 \| \| SINE \| 2208 \| 0 \| 0 \| 0 \| 156054 \| 0.02 \| 154696 \| 0.02 \| \| Other \| 1333525 \| 0.18 \| 1208971 \| 0.17 \| 2980292 \| 0.4 \| 29308813 \| 3.95 \| \| Unknown \| 34896 \| 0 \| 0 \| 0 \| .100723522 \| 13.6 \| 75787119 \| 10.23 \| \| Total \| 112150068 \| 15.14 \| 119516511 \| 16.14 \| 300802992 \| 40.61 \| 365535448 \| 49.35 \|   **Supplemental Table 4 Summary statistics of the annotated transposable elements**  **(TE) in the *O*. *fragrans* genome.** |  |  |  |  |  |  |  |  |
| --- | --- | --- | --- | --- | --- | --- | --- | --- | --- | --- | --- | --- | --- | --- | --- | --- | --- | --- | --- | --- | --- | --- | --- | --- | --- | --- | --- | --- | --- | --- | --- | --- | --- | --- | --- | --- | --- | --- | --- | --- | --- | --- | --- | --- | --- | --- | --- | --- | --- | --- | --- | --- | --- | --- | --- | --- | --- | --- | --- | --- | --- | --- | --- | --- | --- | --- | --- | --- | --- | --- | --- | --- | --- | --- | --- | --- | --- | --- | --- | --- | --- | --- | --- | --- | --- | --- | --- | --- |

**Supplemental Table 5 Summary statistic of the annotated noncoding RNA sequences in *O. fragrans* genome.**

| **Type** | **Copy Number** | **Average Length (bp)** | **Total Length (bp)** | **Percentage (%) of Genome** |
| --- | --- | --- | --- | --- |
| rRNA | 49 | 1677.10 | 82178 | 0.007985 |
| 18S | 13 | 1779.54 | 23134 | 0.002248 |
| 28S | 14 | 3994.21 | 55919 | 0.005434 |
| 5.8S | 15 | 154.80 | 2322 | 0.000226 |
| 5S | 7 | 114.71 | 803 | 0.000078 |
| snRNA | 2058 | 108.23 | 222746 | 0.021644 |
| CD-box | 1840 | 104.96 | 193131 | 0.018766 |
| HACA-box | 66 | 127.74 | 8431 | 0.000819 |
| splicing | 152 | 139.37 | 21184 | 0.002058 |
| miRNA | 525 | 116.98 | 61417 | 0.005968 |
| tRNA | 847 | 74.44 | 63049 | 0.006126 |

**Supplemental Table 6 Summary statistics of the functional genes of *O. fragrans*.**

| **Specie** | **Total numer of gene** | **Average transcript length(bp)** | **Average CDS length(bp)** | **Average exon number per gene(bp)** | **Average exon length(bp)** | **Average intron length(bp)** |
| --- | --- | --- | --- | --- | --- | --- |
| *O.fragrans* | 45,542 | 4,065.24 | 1,142.72 | 5.24 | 2217.92 | 688.66 |
| *A.thaliana* | 27,411 | 1,856.41 | 1,205.11 | 5.09 | 236.78 | 159.26 |
| *O.europaea* | 39,579 | 3,689.11 | 1,163.25 | 4.77 | 243.73 | 669.5 |
| *S.indicum* | 27,072 | 2,816.89 | 1,178.23 | 4.73 | 249.2 | 439.55 |
| *S.tuberosum* | 27,797 | 4,225.06 | 1,278.26 | 5.11 | 250.24 | 717.32 |
| *V.vinifera* | 24,821 | 5,697.52 | 1,343.47 | 5.22 | 257.57 | 1,032.77 |

**Supplemental Table 7 Summary statistic of annotated genes in *O. fragrans* genome. Genes were annotated by the combination of *de novo*, homology-based, and empirical data sets obtained from the full-length transcriptome analyses.**

|  | **Gene set** | **Total number of gene** | **Average transcript length(bp)** | **Average CDS length(bp)** | **Average exons number per gene** | **Average exons length(bp)** | **Average intro length(bp)** |
| --- | --- | --- | --- | --- | --- | --- | --- |
|  | Augustus | 63,912 | 2,089.76 | 871.7 | 4.18 | 208.4 | 382.68 |
| De novo | Geneid | 56,938 | 6,181.04 | 908.26 | 4.79 | 189.58 | 1,390.89 |
|  | Genscan | 38,791 | 10,805.23 | 1,218.99 | 6.49 | 187.88 | 1,746.76 |
|  | Snap | 73.612 | 3,881.48 | 696.58 | 4.31 | 161.65 | 962.47 |
|  | *A.thaliana* | 48,327 | 2,357.62 | 926.58 | 3.43 | 269.89 | 588.2 |
| Homology | *O.europaea* | 32,262 | 2,499.1 | 950.2 | 3.75 | 253.4 | 563.3 |
|  | *S.indicum* | 44,207 | 2,129.39 | 864.78 | 2.99 | 289.36 | 636.0 |
|  | *S.tuberosum* | 31,320 | 2,603.13 | 969.4 | 3.7 | 262.34 | 606.22 |
|  | *V.vinifera* | 41,750 | 2,727.86 | 1,089.88 | 3.63 | 300.14 | 622.55 |
| PASA | Transdecoder | 59,536 | 4,242.07 | 1,235.38 | 5.3 | 232.89 | 689.49 |
| **Final set** | **EVM** | **45,542** |  | **1,142.72** | **5.24** | **217.92** | **688.66** |


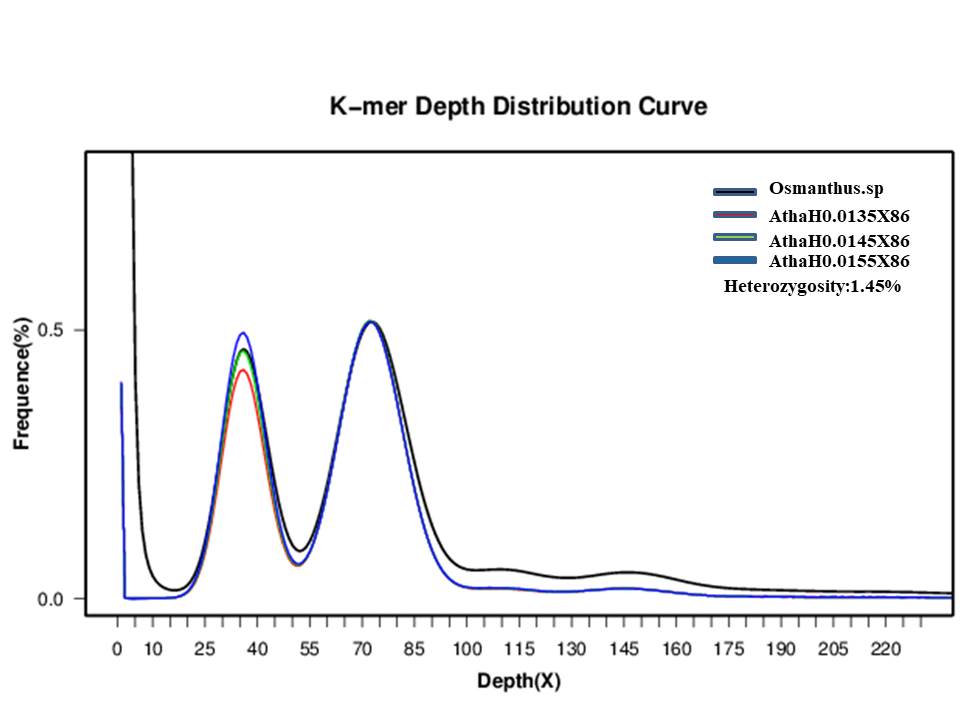
**Supplemental Fig. 1: The distribution of *O*. *fragrans* 17-mers.** The main peak is 73. Secondary peak is 37. The heterozygosity is consistent with the green line. AthaH0.0145X86 means that the depth of the simulated Illumina PE reads of *Arabidopsis thaliana* is 86 with the expected heterozygosity of 1.45 %.


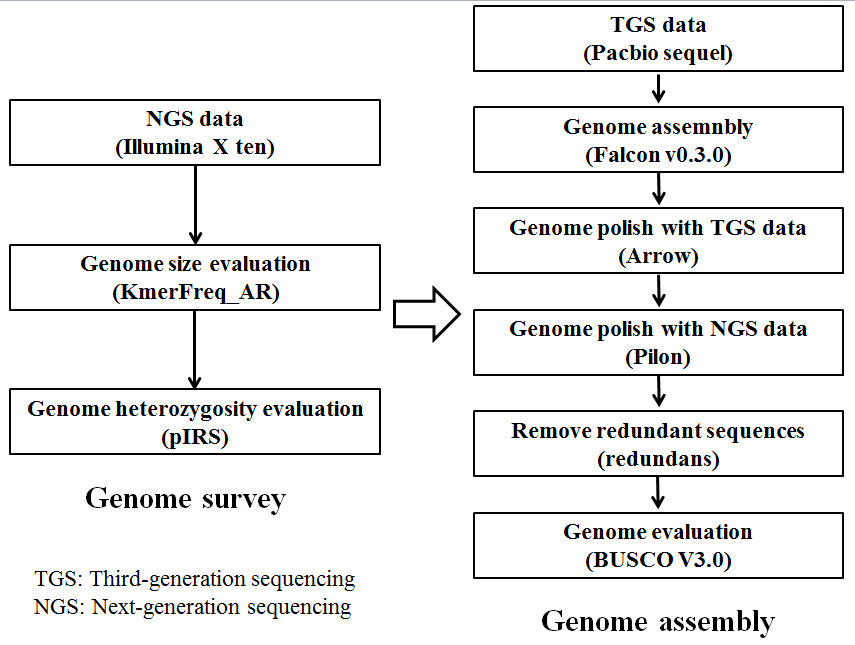
**Supplemental Fig. 2: Integrated work-flow for the assembly of the *O*. *fragrans* genome.**

**
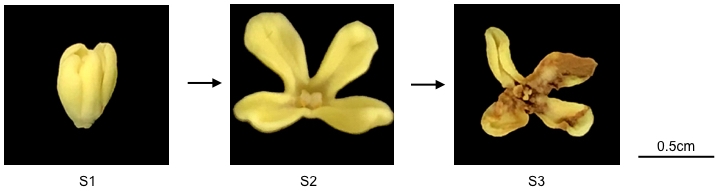
**

**Supplemental Fig. 3: The flowers at different flowering stages.** S1: early, S2: middle, S3: late.
